# Effect of spent turmeric on kidney glycoconjugates in streptozotocin-induced diabetic rats

**DOI:** 10.1186/2251-6581-13-78

**Published:** 2014-08-04

**Authors:** Gurusiddaiah Suresh Kumar, Paramahans Veerayya Salimath

**Affiliations:** 1 Department of Biochemistry and Nutrition, Central Food Technological Research Institute, Mysore 570 020, India; 2 Department of Lipid Science and Traditional Foods, Central Food Technological Research Institute, Mysore 570 020, India

**Keywords:** Diabetic nephropathy, Glycoproteins, Heparan sulfate

## Abstract

**Background:**

Curcumin known to have number of medicinal use and masked the fiber containing ukonan like active polysaccharide in turmeric and its pharmacological effect will be addressed on diabetic nephropathy particularly the glycoconjugates of extracellular components *viz*., glycoproteins and glycosaminoglycans - heparan sulfate (HS).

**Methods:**

Male Wistar rats were maintained on AIN-76 diet containing 10% spent turmeric and were grouped into control and STZ induced diabetes SFC/TFC and SFD/TFD, respectively. Diabetic status was monitored using blood and urine, and at the end, harvested kidneys were used to study the amelioration of glycoprotiens (collagen) and HS by enzymatic digestion, spectrophotometric, hydroxyproline and agarose electrophoretic methods.

**Results:**

In the present study spent turmeric (10%) fed diabetic rats showed improved glomerular filtration rate (50%), kidney enlargement (60%) and other glycoconjugate metabolism in kidney. Increased collagen content in diabetic group was observed by hydroxyproline estimation (24%) and periodic acid-Schiff’s (PAS) staining. Furthermore, elevated activities of enzymes involved in the synthesis and degradation of glycosaminoglycans (GAGs) were significantly lowered in spent turmeric fed diabetic group. Improvement in total GAGs (43%) and sulfate content (18%) followed by fractionation of GAGs using specific enzymes led to HS (28%) in the spent turmeric fed diabetic group, when compared to starch fed diabetic group and was further confirmed by electrophoresis of GAG.

**Conclusion:**

These results clearly indicate beneficial role of spent turmeric in controlling glycoconjugates such as glycoproteins and heparan sulfate related kidney complications during diabetes.

## Introduction

Diabetes is a disease of great concern to many, all over the world and is characterized by chronic hyperglycemia, that may lead to micro-vascular complications, including nephropathy and macro-vascular complications when left uncontrolled [1,2]. Kidney is a vital organ that removes waste products of tissue metabolism through plasma into urine and maintains homeostasis of essential cellular bio-molecules [3]. Defect in kidney leads to deleterious effect on normal physiology *vice versa* long term changes in normal physiology (chronic hyperglycemia) will also affect the normal functioning of kidney and its structure. One such pathological change of kidney in diabetes is decreased renal function that includes glomeruli [4]. During diabetic nephropathy, increase in type IV collagen and decrease in heparan sulfate (HS) are known to take place [5,6]. Type IV collagen is one of the first known constituents of the extracellular matrix (ECM) in glomerular basement membrane that provides a scaffold for other ECM components by its network-like structure [7]. Much of the permselectivity to circulating macromolecules is dependent upon this structure. Glomerular basement membrane (GBM) thickening and altered composition during diabetic nephropathy leads to abnormal filtration [8].

Primary goal in the management of diabetes is to control blood glucose level. Dietary intervention is one of the means to manage diabetes. Besides, emphasis is also being laid on pharmacotherapy. American Diabetic Association has recommended that, the diet should be rich in low calorie carbohydrates such as dietary fibers [9]. Both soluble and insoluble dietary fibers (DF) exert a wide range of physiological effects including hypoglycemic effect when consumed, and their complex physico-chemical properties are responsible for these effects. After they escape digestion in the small intestine, they enter the large intestine, where they become substrate for the intestinal microflora that can degrade many of the non-starch polysaccharides into short chain fatty acids (SCFA) [10]. Beneficial effects of SCFA are well documented [11]. Although, dietary fiber treatment is known to maintain blood glucose level [12], their beneficial effects on diabetic complications particularly nephropathy is not well addressed.

It has been our endeavor to look for beneficial effects of newer sources of fiber such as use of industrial waste, potential in controlling diabetes, which would be an ideal way to make use of waste into health benefit food component. Hence, spent turmeric (leftover residue of curcumin-extracted turmeric) was used in the current study which is byproduct of turmeric industry, where oleoresin is extracted mainly. Spent turmeric is rich in dietary fiber (45%), containing both insoluble fiber (43%) and soluble fiber (2%). In our earlier studies we have shown beneficial effects of spent turmeric on various diabetic parameters [13] and also ameliorated intestinal disaccharidase activities [14]. Beneficial effects on GAGs content and tissue composition of various organs such as liver, lungs, heart and testis of diabetic rats [15] are also reported. In the present investigation, an attempt has been made to study the effect of spent turmeric on glycoproteins (collagen) and HS which are important components of kidney GBM during diabetes.

## Materials and methods

The spent turmeric (*Curcuma longa*) was obtained from Flavors and Essence Industry, Mysore, India. Streptozotocin, *p*-nitrophenyl-*N*-acetyl-β-glucosaminide and *p*-nitrophenyl-β-glucuronide, toludine blue, chondroitin sulfate B and chondroitinase ABC (EC 4.2.2.4) were purchased from Sigma–Aldrich (St. Louis, USA). Dimethylmethylene blue was from Aldrich (Milwaukee, USA). Glucose oxidase/peroxidase (GOD/POD) kit was purchased from Span Diagnostics Limited (Surat, India). All other chemicals used were of analytical grade.

### Animals

Male Wistar rats (OUTB-Wistar IND cftri) weighing around 120g were taken for the study from the Institute’s Animal House. The study had clearance from the Institutional Animal Ethical Committee of CSIR-CFTRI. The rats were grouped into control and diabetic groups. Each group was further sub-divided into starch fed control and spent turmeric fed control (SFC/TFC) and starch fed diabetic and spent turmeric fed diabetic (SFD/TFD). Subgroups had 6 rats each in control groups and 14 rats each in diabetic groups. The rats were maintained on AIN-76 diet [13].

### Preparation of spent turmeric and diet

Spent turmeric obtained was curcumin-free turmeric. It was subjected to acetone extraction (7- 8 h), to remove residual curcumin, if any, and washed with ethanol (95%) and air dried. The starch-fed groups received starch based diet whereas experimental rats were fed with spent turmeric (10%, w/w) replacing starch in AIN-76 diet. These diets were given after inducing diabetes in rats and grouping.

### Induction of diabetes

Diabetes was induced in rats by single intra-peritoneal injection of streptozotocin (STZ) (Sigma, USA) at a level of 55 mg/kg body weight in freshly prepared citrate buffer (0.1M, pH 4.5). Control rats were injected with citrate buffer only. Soon after streptozotocin injection, glucose water (5%) was given for two days [13].

### Collection of urine, blood and kidney samples

Urine was collected under a layer of toluene by keeping the rats in metabolic cages for a period of 24 h. The amount of reducing sugar excreted in the urine was measured one week before sacrificing. Blood was drawn from retro-orbital plexus into tubes containing heparin (20 U/ mL of blood) and from the heart at the time of sacrificing under anesthesia, to measure fasting blood glucose. Kidneys were harvested and weighed at the end of the experimental period (45 days). A small piece of tissue was excised and stored in the fixative (10% formalin), and the rest was stored at -20°C until further analysis.

### Measurement of urine sugar, fasting blood glucose and glomerular filtration rate (GFR)

The reducing sugar content in the urine was measured by DNS method [13]. Blood glucose level was measured using commercially available glucose kit. Creatinine was estimated by Folin’s method in blood and urine (24 h collection under toluene). Glomerular filtration rate (GFR) was determined according to the method of Nandini et al. [16].

### Enzyme activities

Activity of renal enzyme, L-glutamine-fructose-6-phosphate amino transferase (GFAT) was estimated by the method of Pogell and Gryder [17]. Activities of *N*-acetyl-β- glucosaminidase (NAG) and β-glucuronidase were estimated by using PNP derived substrates and the released PNP was estimated [18].

### Preparation of kidney powder, isolation of GAGs and electrophoresis

Kidneys were freed of external fat and enveloped capsule. It was cut into small pieces and dried in acetone at 4°C for about a month by changing acetone every week. Later, the tissue was defatted using petroleum ether in a Soxhlet extraction apparatus. The dried kidney tissue was pulverized thoroughly in a pestle and mortar and was taken up for further analysis. Papain digestion was used for the isolation of GAGs form kidney powder followed by separation on agarose gel electrophoresis and visualized using toluidine blue stain [13].

### Analytical methods

Powdered tissue or glycosaminoglycan samples were taken for estimation after hydrolysis with 2N trifluoro acetic acid for total sugar and uronic acid, 2N HCl for amino sugar and 60% formic acid for sulfates at 100°C in sealed tubes in an oven for 8 h. For protein, samples were solubilized in 0.1M sodium hydroxide solution by repeated sonication and vortexing [16]. Total sugar was estimated by phenol–sulfuric acid method, amino sugar by the method of Ludoweig and Benmaman [19], uronic acid by carbazole method [20], sulfate by turbidometry [21], protein content by Lowry’s method. Sulfated GAG was quantified using dimethylmethylene blue [22] and collagen content by hydroxylproline estimation [23].

### Histopathology

#### Periodic acid-Schiff’s stain (PAS)

The kidneys were kept in fixative (10% formalin) immediately after their removal and sections of 5μm thickness were taken after series of alcohol (70% to 100%) washings and paraffinization. After deparaffinization and hydration, sections were placed in 1% periodic acid for 15 min followed by water wash and Schiff’s reagent treatment, followed by staining with Gills hematoxylin. Images were taken at 40× magnifications and analyzed by assessing the intensity scores between 0 and 5.

#### Statistical analysis

Data obtained was statistically analyzed using Duncan’s multiple range test (DMRT) at significance level (*p <* 0.05).

## Results

Diabetic status was monitored regularly for urine volume, urine sugar and fasting blood glucose and they were increased in SFD group and decreased in TFD group (Table 1). The diabetic symptoms were obvious in the starch fed group (SFD) and mortality initially started in SFD on 45th day onwards and were sacrificed at the end of experimental period under anesthesia.

**Table 1:** Effect of spent turmeric on fasting blood glucose, urine sugar, and urine output in control and diabetic rats

**Groups**	**Fasting blood glucose (mg/dL)**	**Urine sugar (g/day)**	**Urine output (mL/24 h)**	**Glomerular filtration rate (mL/min)**
SFC	108.6 ± 03.1^a^	0.031 ± 0.002^a^	15.0 ± 0.8^a^	0.34 ± 0.03^a^
SFD	351.8 ± 21.3^b^	6.300 ± 0.540^b^	70.8 ± 4.6^b^	7.33 ± 1.47^b^
TFC	108.6 ± 02.5^a^	0.025 ± 0.002^a^	16.5 ± 1.0^a^	0.46 ± 0.04^a^
TFD	205.4 ± 25.1^c^	5.100 ± 0.820^c^	58.5 ± 9.6^c^	3.68 ± 0.12^c^

SFC- Starch fed control; SFD-Starch fed diabetic.

TFC-Spent turmeric fed control; TFD-Spent turmeric fed diabetic.

Values are means ± SE of 6 rats in control and 8 rats in diabetic groups. Means in the same column not sharing a common superscript (a, b, c) are significantly different at *p <* 0.05.

Glomerular filtration rate (GFR) is an important marker of diabetic nephropathy. Creatinine excretion is used as an index of glomerular filtration rate. The GFR increased considerably (>15 fold) in starch fed diabetic group (SFD) compared to the control group (SFC, Table 1). The increased GFR in the starch fed diabetic (SFD) group was ameliorated considerably (50.2%) (*p <* 0.05) in spent turmeric fed diabetic (TFD) group.

### Effect of spent turmeric on kidney weight and its composition in control and diabetic rats

**Figure 1:**
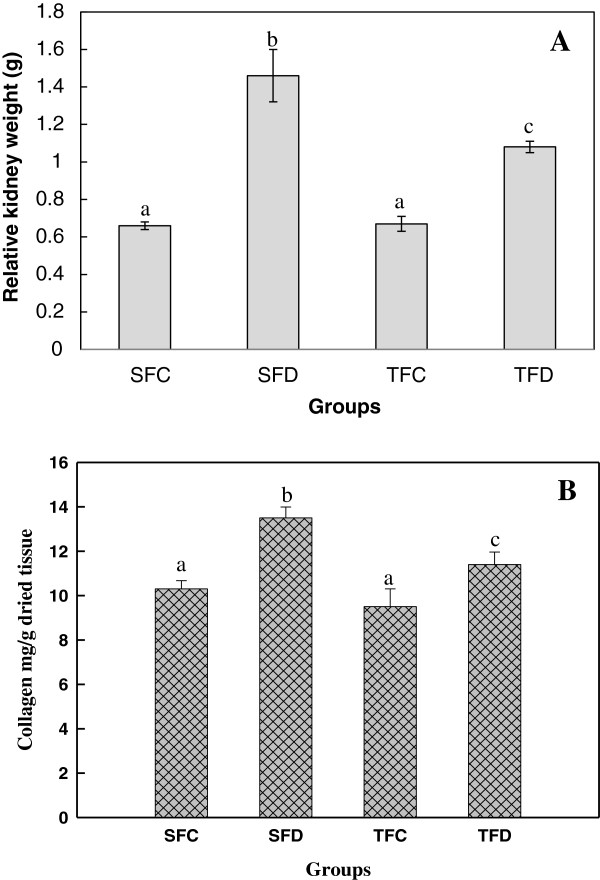
**Effect of spent turmeric on kidney hypertrophy (A) and collagen content (B).** Abbreviations as in Table 1. Values are means ± SE of 6 rats in control and 8 rats in diabetic groups. Means in the bar graph not sharing a common alphabets are significantly different at *p <* 0.05.

Renal enlargement, as indicated by an increase in size in relation to its body weight, was observed in diabetic groups which was approximately twice than that of control. The increase was ameliorated to the tune of 64% by feeding spent turmeric (TFD). Control rats (SFC/TFC) did not show any difference among the groups (Figure 1A).

Glycoconjugate composition of kidney was examined by estimating the contents of total sugar, amino sugar, uronic acid, sulfate and protein and is shown in Table 2. There were no significant differences in the contents of total sugar, amino sugar, uronic acid, sulfate and protein among the control groups (SFC and TFC). But there was minimal, yet significant increase in the contents of total sugar, uronic acid and amino sugar during diabetes (SFD) when compared to control group (SFC) and spent turmeric was found effective in controlling this increase during diabetes in the TFD group. Sulfate content in the total tissue did not show any significant difference between controls, diabetic or treated groups. Content of protein decreased during diabetes (SFD) compared to control group (SFC/TFC). Spent turmeric feeding to diabetic rats showed an increase in the protein content and was statistically insignificant.

**Table 2:** Effect of spent turmeric on the composition of kidney in control and diabetic rats

**Groups**	**Total sugar**	**Amino sugar**	**Uronic acid**	**Sulphate**	**Protein**	**Collagen**
	**mg/g dry tissue**					
SFC	22.05 ± 1.4^a^	5.35 ± 0.1^a^	1.37 ± 0.2^a^	5.68 ± 0.2^a^	661.7 ± 19.2^a^	10.3 ± 0.3^a^
SFD	27.26 ± 0.6^b^	7.05 ± 0.4^b^	2.12 ± 0.6^a^	5.76 ± 0.1^a^	609.1 ± 08.6^b^	13.5 ± 0.4^b^
TFC	23.17 ± 0.7^a^	5.38 ± 0.2^a^	1.47 ± 0.1^a^	5.70 ± 0.1^a^	654.2 ± 26.0^a^	9.5 ± 0.8^a^
TFD	23.07 ± 0.6^a^	5.99 ± 0.1^a^	1.68 ± 0.2^a^	5.96 ± 0.2^a^	639.1 ± 19.2^a^	11.4 ± 0.5^a^

Abbreviations as in Table 1.

Values are means ± SE of 6 rats in control and 8 rats in diabetic groups. Means in the same column not sharing a common superscript (a & b) are significantly different at *p <* 0.05.

### Effect of spent turmeric on renal enzyme activities of glycoconjugate metabolism

Activities of renal enzymes, such as L-glutamine fructose-6-phosphate amino transferase (GFAT) involved in the synthesis of amino sugars and degrading enzymes such as N-acetyl-β-glucosaminidase (NAG) and β-glucuronidase were examined in kidney tissues of different groups. Activities of GFAT (40%) and NAG (60%) were increased in the starch fed diabetic (SFD) group, when compared to starch fed control (SFC) group and were improved in TFD group by 14% and 16%, respectively, which was statistically significant (*p <* 0.05). Activity of β-glucuronidase activity increased in the SFD group, when compared to SFC and was ameliorated in TFD group as shown in Table 3.

**Table 3:** Effect of spent turmeric on activities of some of the renal enzymes in control and diabetic rats

**Groups**	**L-Glutamine fructose-6-phosphate aminotransferase (GFAT)**	**N-Acetyl β-glucosaminidase (NAG)**	**β-glucuronidase**
	**μmoles of product formed/g protein/min**		
SFC	54.96 ± 4.21^a^	2393.00 ± 367^a^	2.35 ± 0.26^a^
SFD	77.09 ± 4.79^b^	3869.00 ± 192^b^	3.92 ± 0.31^b^
TFC	58.78 ± 5.37^a^	2258.00 ± 290^a^	2.20 ± 0.08^a^
TFD	66.58 ± 3.22^c^	3250.00 ± 173^b^	2.68 ± 0.27^a^

Abbreviations as in Table 1.

Values are means ± SE of 6 rats in control and 8 rats in diabetic groups. Means in the same column not sharing a common superscript (a, b & c) are significantly different at *p <* 0.05.

### Effect of spent turmeric on the content of kidney collagen in control and diabetic rats

Type IV collagen, one of the important constituents of the kidney was quantitatively measured in terms of hydroxyproline content. It is one of the characteristic amino acids present in collagen. Hydroxyproline content was increased in SFD group, when compared to its control (SFC), which was partially ameliorated by feeding spent turmeric, and was statistically significant (*p <* 0.05), when compared to starch fed diabetic group (Figure 1B).

### Effect of spent turmeric on kidney histopathology of glycoprotein of control and diabetic rats

**Figure 2:**
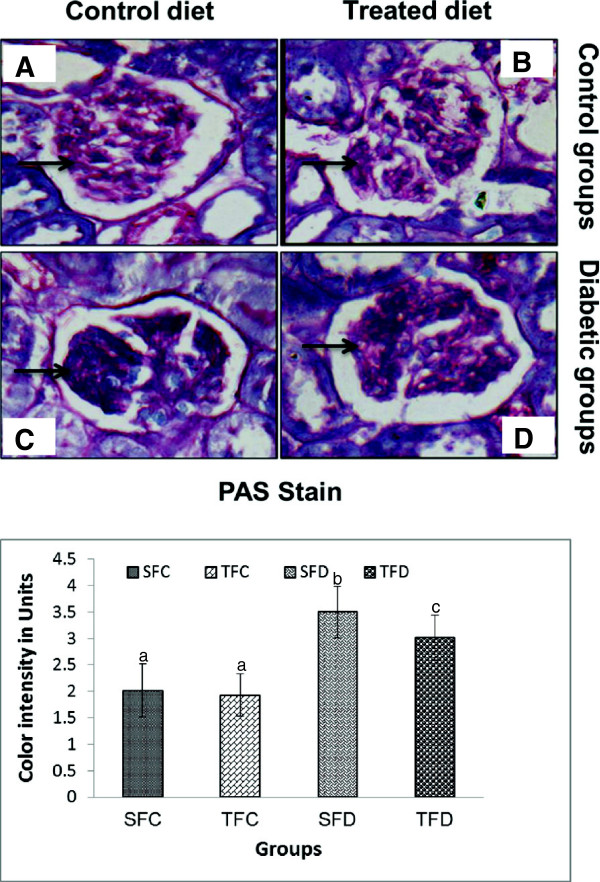
**Effect of spent turmeric on glycoproteins of kidney glomeruli in control and diabetic rats.** Histology of kidney glomeruli sections stained with periodic acid-Schiff (PAS) for control groups **(A, B)** and diabetics groups **(C, D)**, receiving control diet **(A, C)** and treated diet **(B, D)**. Black arrows are showing pink stain for glycoprotein in glomeruli. Graphical representation of colored stains **(E)**. Abbreviations as in Table 1 and Figure 1.

Normally periodic acid-schiff’s stain (PAS) is used to identify glycoproteins by histopathological examination. Here, PAS stain was used to study the effect of feeding, spent turmeric on glycoprotein content in the kidney of diabetic rats and is presented in Figure 2. Results indicated that there is an increase in glycoprotein content, which apparently includes all types of collagen including collagen during diabetes. PAS stained sections were analyzed for color intensity and scored as described in RESEARCH DESIGN AND METHODS: 2.0 ± 0.5 vs. 3.5 ± 0.49 for SFC vs. SFD (*P <* 0.005) (Figure 2B). Likewise spent turmeric fed group reduced their staining uptake (staining score: 3.025 ± 0.42, <0.005 when compared with starch fed diabetic group).

### Effect of spent turmeric on components of glycosaminoglycans of control and diabetic rats

The composition of kidney GAGs, examined by estimating the contents of total sugar, amino sugar, uronic acid and sulfate are presented in Table 4. The content of total sugars in the GAG decreased during diabetes in the starch fed diabetic group (SFD), compared to control (SFC), and amino sugar and uronic acid being the components of GAGs were also decreased. Addition of spent turmeric in the diet of diabetic rats showed improvement and were statistically significant (*P <* 0.05) when compared to diabetic control (SFD). The content of sulfate decreased by about 50% in SFD when compared to SFC and spent turmeric in the diet proved beneficial in preventing the decrease in sulfate content by 18% when compared to SFD.

**Table 4:** Effect of spent turmeric on the composition of kidney GAGs in control and diabetic rats

**Groups**	**Total sugar**	**Amino sugar**	**Uronic acid**	**Sulfate**
	**μg/g dry kidney tissue**			
SFC	9682^a^	928^a^	1354^a^	2521^a^
SFD	3431^b^	604^b^	0715^b^	1363^b^
TFC	9583^a^	895^a^	1495^a^	2385^a^
TFD	4959^c^	683^b^	0831^b^	1670^c^

Abbreviations as in Table 1.

Values are pooled samples of 6 control rats and 8 diabetic rats in groups. Means in the same column not sharing a common superscript (a, b & c) are significantly different at *p <* 0.05.

### Effect of spent turmeric on kidney total sulfated GAG, heparan sulfate and chondroitin sulfate in renal tissue of control and diabetic rats

**Figure 3:**
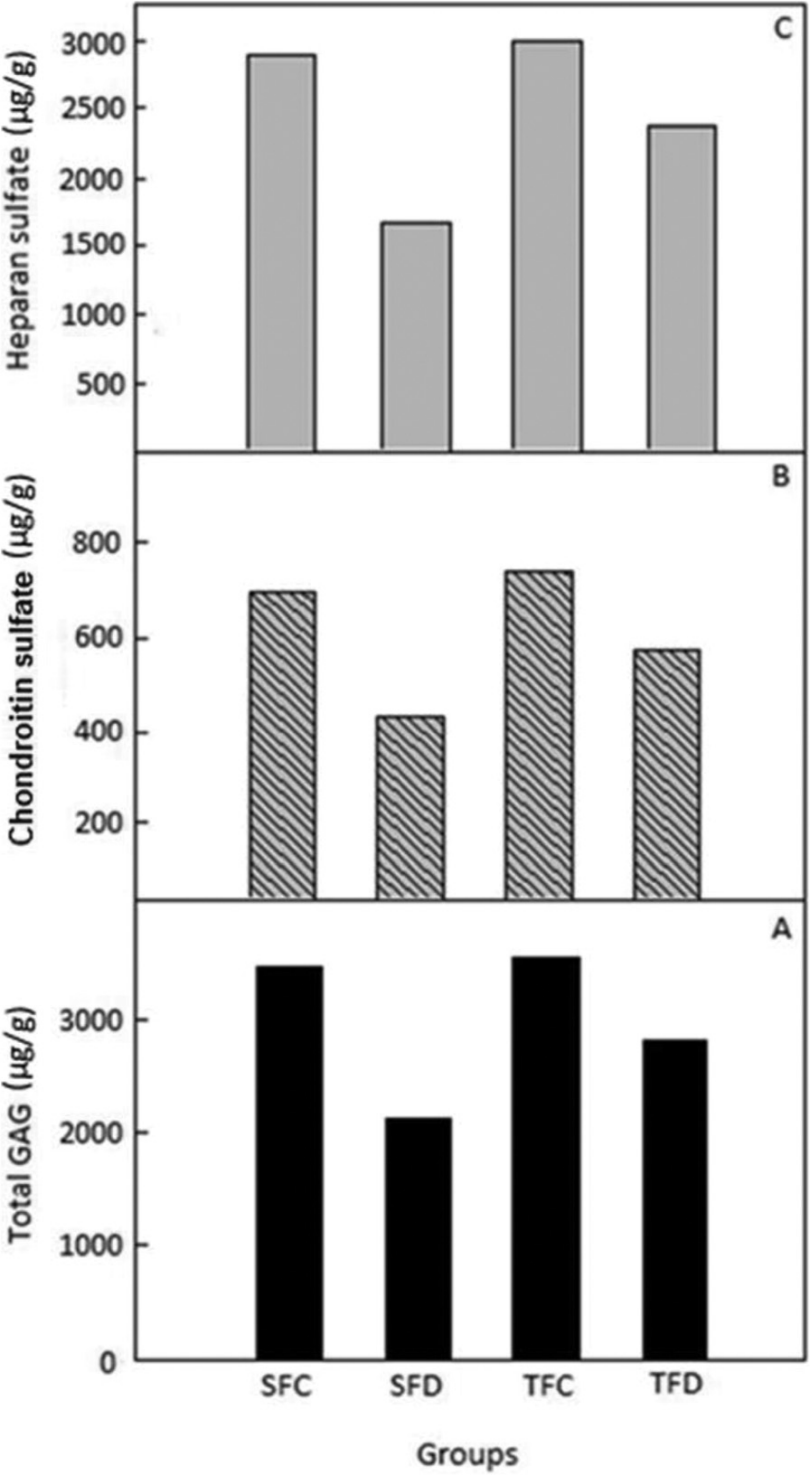
**Effect of spent turmeric on sulphated glycosaminoglycans (GAGs) of renal tissue in control and diabetic rats. A** - Total GAG, **B** - Heparan Sulphate, **C** - Chondroitin Sulpahte. Values are the average of triplicates of 6 pooled samples in control and 8 pooled samples in diabetic rats.

Content of sulfated GAG in the isolated GAG samples of controls (SFC and TFC) showed approximately equal amount of GAG in the kidney and was significantly decreased (39%) in SFD group. Decreased sulfated GAG was found to be improved by 24% in the spent turmeric fed group (Figure 3A), when compared to SFD group. Furthermore, obtained GAGs were fractionated based on their specificity to chondroitinase ABC. The content of chondroitin sulfate was also decreased in the starch fed diabetic group (SFD) and was improved in TFD group by 27% (Figure 3B). The undigested fraction represented the HS. The content of HS decreased by 43% in the starch fed diabetic group (SFD) compared to its control (SFC) and spent turmeric fed group was effective in controlling HS decrement to 28% in the diabetic kidney (Figure 3C).

### Electrophoretic profile of kidney glycosaminoglycans of control and diabetic rats

**Figure 4:**
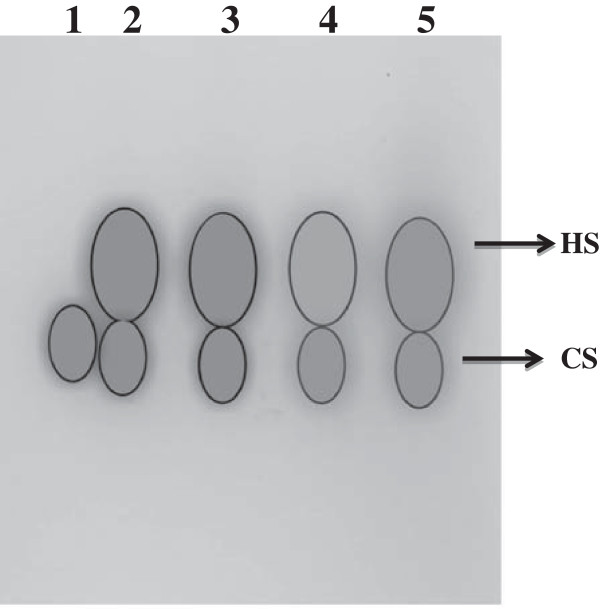
**Agarose gel electrophoresis of glycosaminoglycans (GAGs) isolated from control, diabetic, and spent turmeric treated rat kidneys.** Lane 1. Chondroitin sulphate B (sigma); Lane 2. Starch fed control; Lane 3.Spent turmeric fed control; Lane 4. Starch fed diabetic; Lane 5. Spent turmeric fed diabetic; HS. Heparan sulfate; CS. Chondroitin Sulphate.

**Figure 5:**
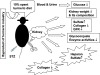
**Mode of actions of spent turmeric in amelioration of diabetic nephropathy.** The figure depicts beneficial effect of feeding spent turmeric on diabetes and diabetic nephropathy kidney profiling for glycoconjugate showing beneficial effect on heparan sulfate and collagen, and glycoconjugate metabolizing enzymes.

Separation of total GAG was achieved by agarose gel electrophoresis upon loading the normalized GAG samples of equal amount of kidney. Heparan sulfate was the major glycosaminoglycan and the control samples (SFC and TFC) showed approximately equal amounts of heparan sulfate (Figure 4, Lane 2 and 3). Decreased heparan sulfate in the starch fed diabetic group (SFD) was clearly ameliorated by TFD group (Figure 4, Lane 4 and 5) and chondroitin sulfate B was used as standard. Mode of actions of spent turmeric is depicted in Figure 5. Normal glucose level both in blood and urine, reduced GFR, kidney hypertrophy and enzymes involved in the glycoconjugate metabolism for appropriate synthesis of heparan sulphate and type IV collagen present in ECM were altered during diabetes and were ameliorated by feeding with spent turmeric (10%).

## Discussion

The present study focuses on the effect of diabetes on glycoconjugates of kidney with particular emphasis on glycoproteins (collagen) and GAGs (HS) and its modulation by spent turmeric. In earlier stages of diabetes mellitus, a hypertrophy and hyper-function of the kidney was observed by Christiansen et al. [24]. By feeding spent turmeric, both GFR (50%) and kidney enlargement (60%) were reduced. Increase in relative weight of the kidney during diabetes has been reported by others. The degree of renal enlargement was correlated with the degree of glycemic control [25]. Curcumin, the active principle in turmeric, was more effective in ameliorating diabetic condition than whole turmeric (*Curcuma longa*) and attenuated kidney enlargement in streptozotocin-induced diabetic rats [26,27]. However, the polysaccharide from *Curcuma longa* includes ukonan A, ukonan B, ukonan C, and ukonan D and they exhibit immunomodulatory effect [28,29]. Hence, there may be possibility that the polysaccharides of spent turmeric improve the renal function to various extents.

The increase in total sugar, uronic acid and amino sugar content during diabetes may be due to non-enzymatic glycation of tissue proteins and addition of small-sized GAGs [30,31], respectively. Gupta et al. [32] have shown that decrease in soluble proteins in kidney may be due to increase in gluconeogenesis during diabetes.

Increase in expression of L-glutamine-fructose-6-phosphate amino transferase (GFAT) involved in amino sugar synthesis and thickening of glomerular basement membrane and it is the rate-limiting enzyme for high glucose-induced TGF-β expression [33]. It is a transcription factor for the synthesis of ECM proteins and inhibitors for matrix degrading proteins [34]. The levels of GAG degrading enzymes, NAG and β-glucuronidase activity in diabetes can be related to vascular complications and degree of the disorder of glucose metabolism [35]. Katz et al. [36] reported high activity of heparanase, an endo β-glucuronidase during diabetes, which participates in the turnover of glomerular heparan sulfate proteoglycans. Spent turmeric in the diet was effective in preventing the increased activities of glycoconjugate related enzyme activities during diabetes.

Increased synthesis of extracellular matrix components and reduction in the charge density of proteoglycans occurs that eventually leads to proteinuria and glomerular sclerosis [37-39]. The thickening of GBM is also an early feature of diabetic nephropathy. Earlier it is shown that, non-enzymatic glycation of collagen and over expression of TGF-β contribute to GBM thickening [40]. Alterations that occur in glomerular basement membrane, involve lysine or its derivatives, hydroxylysine and glycosylated hydroxylysine. Both lysine and hydroxylysine participate in the peptide chain formation in collagen and elastin [41]. The increase in glycosylation of hydroxylysine would reduce the availability of this amino acid to participate in their formation. Such a defect in cross-linking and bulky carbohydrate substituents on the packing of the peptide chains would contribute to the increased permeability of the basement membrane seen in diabetic nephropathy. Studies have shown that diabetic glomeruli have greater hydroxyproline content than non-diabetic ones [42]. Our results demonstrated increase in PAS-positive material as well as collagen (hydroxylysine) content during diabetes and were ameliorated by spent turmeric.

Hyperglycemia, also results in increase in the activity of PKC-β and TGF-β, thereby increasing collagen and other glycoconjugates in mesangial cells and glomerular basement membrane [40]. This is amply clear in starch fed group with high florescence for collagen which was attenuated in the spent turmeric fed diabetic group.

The main GAG constituent in the glomerular basement membrane is HS (90%) and remaining 10% includes other ECM constituents such as condroitin sulfate, dermatan sulfate and hyaluronic acid. The presence of sulfate groups and carboxyl groups are responsible for the charge density in the lamina rara of GBM. These anionic sites prevent clogging of the basement membranes by circulating plasma macromolecules [43]. Brown et al. [44] have reported reduction in HS content during diabetes in both glomerular basement membrane and our results are in agreement. It was observed that, addition of non-absorbable fibre to the diet of genetically diabetic mice improved glycemic control and retarded the development of diabetic nephropathy [45]. The results obtained by fractionation of GAG’s and also by agarose gel electrophoresis confirmed that, spent turmeric feeding clearly, improved total sulfated GAGs and HS. Decreased HS in agarose electrophoresis was also observed previously [16,46] and our results correlated with those reports.

Improvement of glucose levels by spent turmeric [13] could be one of the factors attenuating the synthesis of collagen and increasing the content of HS. Fermentation of dietary fibres by colon microflora might also be playing a role on these beneficial effects. It is well known that the fermented products of dietary fibre including propionate and butyrate have been implicated in various biological activities [46]. n-Butyrate, in particular is shown to down regulate the PKC-β [47] which may combat the increased synthesis of glycoproteins and improves HS synthesis.

Our earlier studies on n-butyrate had shown the importance of fibres on the activity of intestinal disaccharidases which further facilitated slow absorption of glucose [11,48].

In conclusion, present study demonstrates that, spent turmeric, a byproduct of turmeric industries exerts their effect on diabetic nephropathy particularly glycoproteins and HS of the extracellular matrix in kidney. Elucidated mechanisms will be helpful in further understanding the role of dietary principles in ameliorating some of the pathological conditions associated with diabetes. Thus, spent turmeric which is a good source of dietary fiber is beneficial in the management of diabetes and diabetic nephropathy.

## Competing interests

The authors declare that they have no competing interests.

## Authors’ contributions

GSK participated in the design, study, data acquisition and drafting manuscript, PVS also participated in the design and data analysis. Both authors read and approved the final manuscript.
